# Intracorporeal stapled versus extracorporeal hand-sewn anastomosis in minimal-invasive right hemicolectomy with complete mesocolic excision – a retrospective single center analysis

**DOI:** 10.1007/s00423-025-03749-x

**Published:** 2025-06-09

**Authors:** Maximilian Brunner, Katja Bondartschuk, Axel Denz, Georg F. Weber, Robert Grützmann, Christian Krautz

**Affiliations:** Department of General and Visceral Surgery, Friedrich-Alexander-University, Krankenhausstraße 12, 91054 Erlangen, Germany

**Keywords:** Right hemicolectomy, Intracorporeal anastomosis, Extracorporeal anastomosis, Postoperative recovery

## Abstract

**Background:**

Minimally invasive right hemicolectomy can be performed with either an extracorporeal or intracorporeal anastomosis, with the latter gaining increasing popularity. This study aimed to evaluate the impact of the anastomotic technique on postoperative outcomes and recovery.

**Methods:**

We retrospectively reviewed 177 patients who underwent minimally invasive right hemicolectomy with complete mesocolic excision (CME) at our institution from 2016 to May 2024. Of these, 96 patients received an extracorporeal hand-sewn end-to-end anastomosis, while 81 patients underwent an intracorporeal stapled side-to-side isoperistaltic anastomosis. The impact of the anastomotic technique on postoperative outcomes and recovery was assessed using uni- and multivariate analyses.

**Results:**

Patients with intracorporeal anastomoses experienced significantly fewer surgical site infections (0% vs. 3%, *p* = 0.032), less postoperative pain at rest and under stress on postoperative day (POD) 4 (*p* = 0.028 and *p* = 0.007, respectively), earlier first bowel movement (POD 2 vs. POD 3, *p* = 0.014) and shorter postoperative hospital stays (5 vs. 6 days, *p* = 0.049). There were no significant differences between the groups in overall morbidity, reoperations or anastomotic leakage rates. Multivariate analysis indicated that the intracorporeal anastomosis technique was significantly associated with enhanced postoperative recovery (defined as first stool by POD 2, full meal tolerance by POD 4 and discharge by POD 6; OR 0.5 [0.2–0.9], *p* = 0.036).

**Conclusion:**

Intracorporeal stapled side-to-side anastomosis may enhance postoperative recovery after minimal-invasive right hemicolectomy with CME.

## Introduction

In recent years, minimally invasive techniques have gained widespread acceptance in colorectal surgery due to their numerous advantages, including reduced postoperative pain, shorter hospital stays and quicker recovery times [1–3]. Right hemicolectomy, a standard procedure for treating various conditions such as colon cancer, Crohn’s disease and diverticulitis, is no exception [4, 5]. One of the key technical aspects of minimally invasive right hemicolectomy is the creation of the anastomosis, which can be performed either intracorporeally or extracorporeally.

Intracorporeal anastomosis (ICA) involves the construction of the anastomosis entirely within the abdominal cavity using laparoscopic or robotic techniques, while extracorporeal anastomosis (ECA) requires externalizing the bowel through a small incision to complete the anastomosis outside the body. Each approach has its proponents and specific advantages, with ongoing debate regarding which method yields better outcomes [6–15].

Proponents of intracorporeal anastomosis argue that it allows for smaller incisions, better bowel mobilization and more precise surgical control, potentially leading to reduced postoperative pain, faster recovery and lower rates of complications such as incisional hernias. On the other hand, extracorporeal anastomosis is a more familiar technique for many surgeons and may reduce operative time while also simplifying certain aspects of the procedure.

The choice between intra- and extracorporeal anastomosis in minimally invasive right hemicolectomy remains a topic of interest in surgical practice [6–15]. This study aims to explore the key differences between these two techniques, focusing on their respective outcomes in terms of operative time, postoperative recovery and complication rates. Understanding the benefits and potential drawbacks of each approach can help guide surgical decision-making and ultimately improve patient outcomes.

## Materials and methods

We retrospectively analyzed 177 consecutive patients who underwent minimally invasive right hemicolectomy with complete mesocolic excision (CME) between 2016 and May 2024 at the University Hospital Erlangen. Patients who had non-oncological resections or open surgical approaches were excluded (*n* = 289). The primary indications for surgery were malignancies, with some cases involving premalignant lesions requiring oncological resection.

Data were collected on patient demographics, comorbidities, preoperative parameters, intraoperative findings and the postoperative course, including morbidity and were compared between patients who underwent extracorporeal anastomosis (ECA) and intracorporeal anastomosis (ICA) during minimally invasive right hemicolectomy with complete mesocolic excision (CME). No randomization was performed. There was no predefined system for assigning the type of anastomosis. The decision was made by the operating surgeon based on intraoperative findings and personal preference.

The primary endpoints of the study were short-term postoperative outcomes, including morbidity, recovery and postoperative pain. Postoperative pain was assessed using the Visual Analog Scale (VAS), while surgical site infections (SSI) were classified according to the CDC definition for superficial incisional SSIs [16]. For the multivariate analysis, a composite parameter termed “enhanced recovery” was created, defined by the following criteria: first bowel movement by at least postoperative day (POD) 2, a full meal plan by at least POD 4, and discharge by at least POD 6.

### Surgical techniques

Preoperative bowel preparation for all patients included mechanical bowel cleansing with Clean Prep. All surgeries were performed minimally invasively under general anesthesia by two experienced colorectal surgeons specialized in minimally invasive surgery, following a standardized oncological technique with CME [17]. The choice of surgical approach (robotic vs. laparoscopic) was primarily determined by the availability of the robotic system. The choice of anastomotic technique was made by the operating surgeon based on intraoperative findings and personal preference. Initially, extracorporeal anastomosis was the preferred approach; however, a gradual shift toward intracorporeal anastomosis occurred over the course of the study period.

**Fig. 1:**
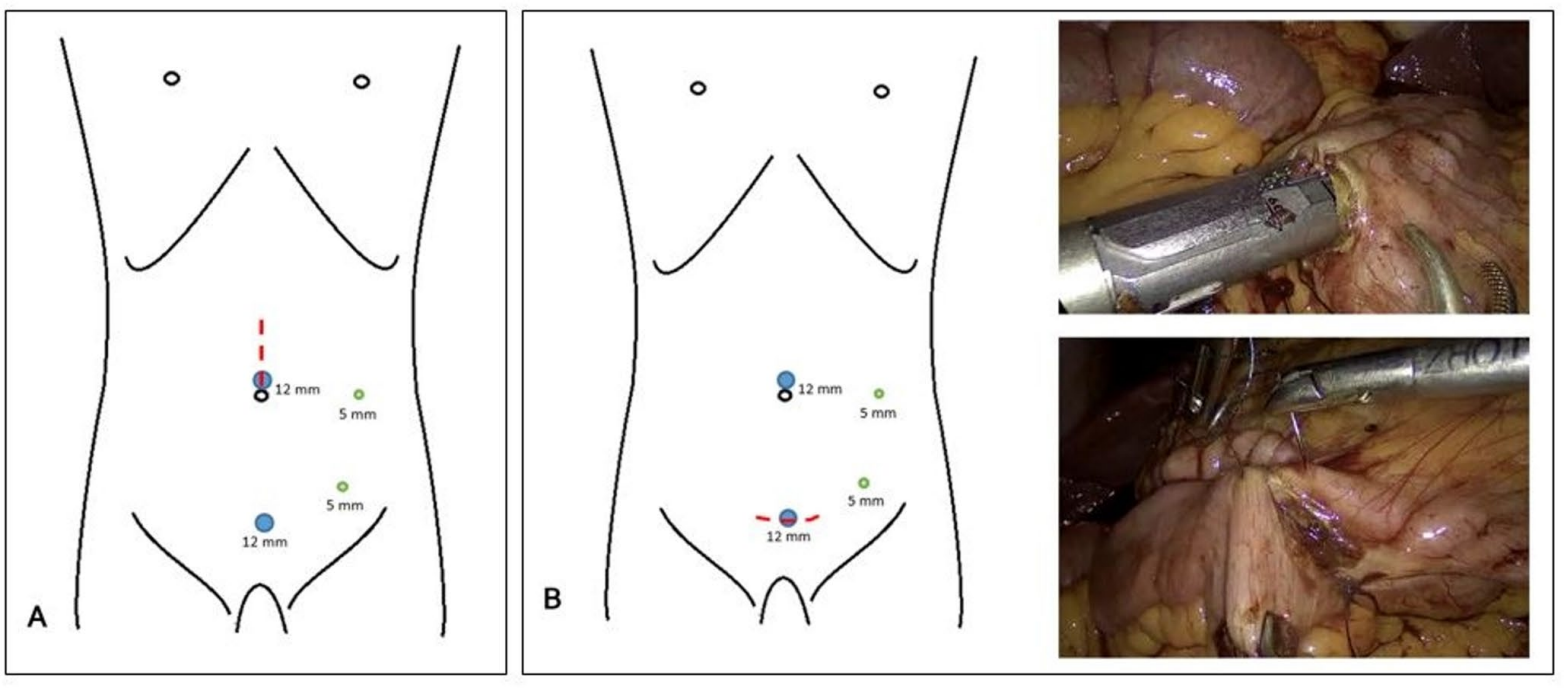
Port placement and surgical approach of extracorporeal (**A**) and intracorporeal (**B**) anastomosis during minimal-invasive right hemicolectomy with CME

For the extracorporeal anastomosis, a median supraumbilical abdominal wall incision (approximately 5–8 cm) was made to extract the specimen, including the terminal ileum and transverse colon. The abdominal wall was protected using a wound protector (Alexis, Applied Medical). Outside the abdomen, the terminal ileum and transverse colon were transected and an end-to-end isoperistaltic hand-sewn continuous anastomosis was performed using either a one-row or double-row technique with 4 − 0 or 5 − 0 PDS sutures (Fig. 1).

**Fig. 2:**
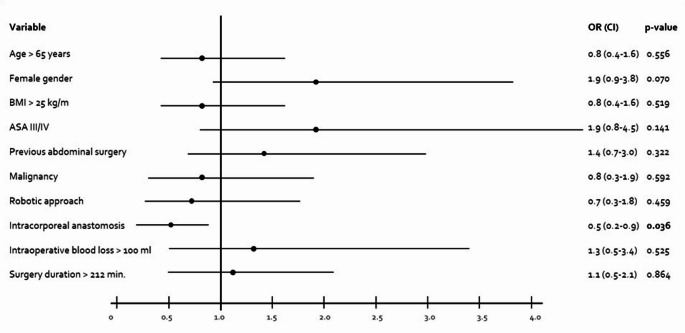
Forrest plot displaying multivariate regression for fail of enhanced recovery (defined as first stool at least on POD 2, complete meal plan at least on POD 4 and discharge at least on POD 6); POD = Postoperative day; OR = Odds Ratio; BMI = Body Mass Index; ASA = American Society of Anesthesiologists

For the intracorporeal anastomosis, the terminal ileum and transverse colon were transected intracorporal with a linear stapler. The specimen was removed via a suprapubic incision using a wound protector (Alexis, Applied Medical). A side-to-side isoperistaltic anastomosis was then created between the terminal ileum and transverse colon. Two single sutures with 3 − 0 Vicryl were placed to approximate the bowel ends. Enterotomies were performed on both sides of the bowel and the intracorporeal anastomosis was stapled with a linear stapler (60 mm blue cartridge). The enterotomy was closed by continuous suturing, using 3 − 0 Vicryl for the dorsal side and 4–0 V-Loc (Medtronic) for the ventral side (Fig. 1).

### Postoperative management

Prophylaxis for postoperative nausea and vomiting (PONV) was administered only in patients with a positive history. The nasogastric tube was routinely removed immediately after surgery. The use of intra-abdominal drains was based on the intraoperative assessment and discretion of the operating surgeon. Drains were typically removed early, most often on postoperative day 2 or 3, depending on drainage volume and quality. Pain management followed a standardized protocol with metamizole and, if necessary, escalation according to the WHO analgesic ladder. Early oral intake was initiated as tolerated. Patients were discharged once they tolerated a full diet, demonstrated regular bowel movements and adequate pain control was achieved.

### Statistical analysis

Data analysis was performed with SPSS software (SPSS, version 24.0). Comparisons of metric and ordinal data were calculated with the Student’s t-test or Mann Whitney U test. The Chi-square test was used for categorical data. Statistical significance was set at *p* < 0.05. Potential factors influencing enhanced recovery (as defined above) were analyzed using multivariate analysis.

## Results

### Demographics

Of the 177 patients (mean age: 65 years, 51% female) who underwent right hemicolectomy with CME, 96 patients received an ileotransversostomy by extracorporal anastomosis (ECA group) and 81 patients by intracorporal anastomosis (ICA group). Demographic data of the two groups including age, gender BMI, comorbidities, tumor location and neoadjuvant treatment as well as preoperative blood results did not differ significantly between the two groups and are shown in Table 1.

**Table 1:** Patient demographics

	All patients *n* = 177	ECA *n* = 96	ICA *n* = 81	*p*-value
Mean age (years), median (IQR)	65 (23)	65 (18)	66 (24)	0.534
Sex, n (%)				0.131
Male Female	86 (49) 91 (51)	52 (54) 44 (46)	34 (42) 47 (58)	
BMI (kg/m^2^), median (IQR)	25.3 (6.2)	25.0 (6.2)	25.6 (6.3)	0.749
ASA, n (%)				0.065
1 2 3 4	10 (6) 122 (69) 42 (24) 3 (2)	8 (8) 70 (73) 17 (18) 1 (1)	2 (3) 52 (64) 25 (31) 2 (3)	
Immunosuppression/steroid therapy, n (%)	14 (8)	7 (7)	7 (9)	0.785
Comorbidities, n (%)				
Arterial hypertension Cardiac disease Diabetes mellitus	84 (48) 33 (19) 24 (14)	45 (47) 21 (22) 15 (16)	39 (48) 12 (15) 9 (11)	0.881 0.251 0.509
Prior abdominal surgery, n (%)	67 (38)	39 (41)	28 (35)	0.440
Neoadjuvant chemotherapy, n (%)	2 (1)	1 (1)	1 (1)	1.000
Tumor localization, n (%)				0.340
Terminal ileum/appendix/cecum Ascending colon Right flexure/right transverse colon	82 (46) 76 (43) 19 (11)	41 (43) 46 (48) 9 (9)	41 (51) 30 (37) 10 (12)	
Preoperative blood results, median (IQR)				
White blood cell count (x 10^9^/l) Hemoglobin (g/dl) CRP (mg/l) Creatinine (mg/dl) Albumin (g/l) (*n* = 78)* CEA (ng/ml) (*n* = 91)*	6.8 (2.8) 12.9 (3.4) 3 (6) 0.8 (0.2) 42.2 (5.0) 2.0 (4.0)	6.5 (3.1) 13.1 (3.1) 3 (6) 0.8 (0.2) 42.0 (6.0) 1.7 (4.0)	6.8 (2.6) 12.4 (3.4) 3 (6) 0.8 (0.2) 42.4 (4.0) 2.3 (4.0)	0.955 0.311 0.482 0.557 0.738 0.693

*ECA* Extracorporeal anastomosis, *ICA* Intracorporeal anastomosis, *IQR* Interquartile range, *BMI*, Body Mass Index, *ASA* American Society of Anesthesiologists, *CRP* C-reactive protein, *CEA* Carcinoembryonic antigen

*not always determined

### Surgical parameters

The majority of patients (78%) underwent surgery due to a malignant lesion in the right colon (mostly pT2/3 (55%) pN0 (75%) tumors), in 22% of the patients a premalignant lesion indicated surgery. The most common used approach was laparoscopic (86%) and robotic in 14%. ICA was significantly more often performed during the robotic approach compared to the laparoscopic approach (84% vs. 39%, *p* < 0.001). Median operative time was 212 min. In all except of one patients R0-resection could be reached (Table 2).

**Table 2:** Surgical and pathological details

	All patients *n* = 177	ECA *n* = 96	ICA *n* = 81	*p*-value
Indication for surgery, n (%)				0.141
Malignant lesion Premalignant lesion	139 (78) 38 (22)	71 (74) 25 (26)	68 (84) 13 (16)	
Surgical approach, n (%)				**< 0.001**
Laparoscopic Robotic	152 (86) 25 (14)	92 (96) 4 (4)	60 (74) 21 (26)	
Operative time (min.), median (IQR)	212 (68)	210 (75)	212 (58)	0.817
Intraoperative blood loss (ml), median (IQR)	50 (50)	50 (50)	50 (50)	0.918
T category (*n* = 139), n (%)				0.496
pT1 pT2/3	62 (45) 77 (55)	34 (48) 37 (52)	28 (41) 40 (59)	
Lymph node retrieval, median (IQR)	25 (13)	24 (12)	26 (14)	0.550
N category (*n* = 139), n (%)				0.171
pN0 pN+	104 (75) 35 (25)	57 (80) 14 (20)	47 (69) 21 (31)	
Resection margin, n (%)				1.000
R0 R1	176 (100) 1 (1)	95 (99) 1 (1)	81 (100) 0 (0)	

*ECA* extracorporeal anastomosis, *ICA* intracorporeal anastomosis, *IQR* Interquartile range

### Postoperative outcome

Morbidity occurred in 24% of all patients being mostly minor complications (56%) and included anastomotic leakage in 2%, postoperative ileus/subileus in 2% and surgical site infections in 3% of all patients. Two patients died postoperatively - one due to an anastomotic leak and one due to postoperative pneumonia in combination with a myocardial infarction (Table 3). Five patients experienced life-threatening complications requiring intensive care treatment (Clavien-Dindo grade IV). These included one case each of anastomotic leak, postoperative pancreatitis, pulmonary embolism, postoperative pneumonia and cardiac arrhythmia.

**Table 3:** Outcome parameters

	All patients (*n* = 177)	ECA (*n* = 96)	ICA (*n* = 81)	*p*-value
Morbidity, n (%)	43 (24)	27 (28)	16 (20)	0.221
Clavien-Dindo, n (%)				0.367
I II III IV V	9 (5) 17 (10) 10 (6) 5 (3) 2 (1)	7 (7) 10 (10) 4 (4) 4 (4) 2 (2)	2 (2) 7 (9) 6 (7) 1 (1) 0 (0)	
Anastomotic leakage, n (%)	3 (2)	2 (2)	1 (1)	1.000
Postoperative ileus/subileus, n (%)	3 (2)	3 (3)	0 (0)	0.251
Superficial surgical site infection, n (%)	6 (3)	6 (6)	0 (0)	**0.032**
Re-surgery, n (%)	7 (4)	5 (5)	2 (3)	0.456
First stool (POD), median (IQR)	2 (1)	3 (2)	2 (1)	**0.014**
First meal (POD), median (IQR)	1 (0)	1 (0)	1 (1)	0.071
Completed meal plan (POD), median (IQR)	4 (2)	4 (2)	4 (2)	0.930
Postoperative pain (VAS) at rest, mean (SD)				
POD 1 POD 2 POD 3 POD 4	1.9 (1.9) 1.3 (1.6) 0.9 (1.3) 0.8 (1.4)	1.8 (1.7) 1.3 (1.4) 1.0 (1.4) 1.0 (1.6)	2.0 (2.1) 1.2 (1.8) 0.8 (1.2) 0.6 (1.2)	0.669 0.282 0.583 **0.028**
Postoperative pain (VAS) under stress, mean (SD)				
POD 1 POD 2 POD 3 POD 4	3.0 (2.2) 2.4 (2.1) 1.6 (1.7) 1.5 (1.9)	3.1 (2.2) 2.6 (2.0) 1.8 (1.8) 1.8 (1.9)	2.8 (2.2) 2.2 (2.1) 1.3 (1.6) 1.1 (1.7)	0.197 0.159 0.082 **0.007**
Length of postoperative hospital stay (days), median (IQR)	6 (2)	6 (2)	5 (3)	**0.049**

*ECA* extracorporeal anastomosis, *ICA* intracorporeal anastomosis, *POD* Postoperative day, *IQR* Interquartile range

Resurgery was needed in 4% of the patients. In three cases, the indication was an anastomotic leak, which was managed by the creation of a stoma. One patient suffered from a small bowel perforation, which was treated with primary suture repair. Another patient developed postoperative pancreatitis requiring surgical drainage. In two patients, relaparoscopy was performed due to elevated inflammatory markers and a clinically suspicious abdomen; however, no intra-abdominal pathology was identified.

Regarding the morbidity parameters only the surgical site infection rate significantly differed between the two groups benefiting the ICA (ICA: 0% vs. ECA: 6%, *p* = 0.032) (Table 3). The mean length of postoperative hospital stay was 6 days being in the ICA group significantly shorter (ICA: 5 days vs. ECA: 6 days, *p* = 0.049).

Postoperative recovery parameter (first stool, first meal, completed meal plan, postoperative pain) are shown in Table 3. Patients with ICA showed a significant shorter duration until the first stool occurred (ICA: 2 days vs. ECA: 3 days, *p* = 0.014) and significant lower pain level on postoperative day 4 (at rest: ICA: 0.6 vs. ECA: 1.0, *p* = 0.028 and under stress: ICA: 1.1 vs. ECA: 1.8, *p* = 0.007) .

### Multivariate analysis for enhanced recovery (defined as first stool at least on POD 2, complete meal plan at least on POD 4 and discharge at least on POD 6)

Including age, gender, BMI, ASA score, presence of previous abdominal surgeries and of malignancy, the surgical approach, the kind of anastomosis, the intraoperative blood loss and the surgical duration in a multivariate analysis as potential influence factors for the postoperative recovery, only the use of an intracorporal anastomosis was identified as independent impact on enhanced recovery (OR0.5 (CI 0.2–0.9), *p* = 0.036) (Fig. 2).

## Discussion

The current study provides evidence supporting the advantages of intracorporeal anastomosis (ICA) over extracorporeal anastomosis (ECA) in minimally invasive right hemicolectomy with CME, particularly in terms of faster postoperative recovery, quicker pain reduction, and lower surgical site infection (SSI) rates. These findings align with and contribute to a growing body of research highlighting the benefits of ICA [6–15].

While the findings support ICA as a potentially superior approach, several limitations warrant consideration. The retrospective design of this study limits causal inferences and although various demographic and clinical factors were controlled for, residual confounding cannot be entirely ruled out. Additionally, while this study provides valuable short-term outcomes, long-term follow-up is necessary to determine whether ICA’s benefits are sustained over time or if any delayed complications arise. The study’s setting in a single institution with a standardized protocol may also limit broader applicability; variations in surgeon experience and patient characteristics across institutions could influence outcomes, indicating a need for multicenter studies to confirm these findings’ generalizability. Furthermore, the potential influence of other factors, such as the type of extraction site incision - which varied between the two anastomotic techniques - cannot be excluded.

The results of our study are well-supported by the mechanisms involved: ICA appears to be less traumatic, allowing for faster bowel function recovery. This accelerated recovery may be due to the reduced manipulation of the abdominal wall and potentially lower inflammatory response with ICA, as it avoids both the larger extraction site and the mesenteric tension required for ECA. Additionally, the reduced SSI rate could be linked to ICA’s minimized need for exteriorization of bowel segments, reducing exposure to external contaminants.

Our study further confirms that the overall complication rate, including anastomotic leakage, is not significantly affected by the choice of anastomosis technique [6–15]. Unlike previous studies that reported a longer operative time for intracorporeal anastomosis, our findings did not show any significant difference in operative time between ICA and ECA [13].

The composite “enhanced recovery” parameter also underscores the advantages of ICA, with ICA patients demonstrating shorter hospital stays. This result highlights ICA’s potential to expedite key postoperative milestones, reinforcing its value in enhanced recovery after surgery (ERAS) protocols, which are increasingly utilized in colorectal surgery [9, 18].

Cost-effectiveness is another important consideration. Reduced hospital stays benefit patients by decreasing their postoperative burden and also have economic implications, potentially lowering healthcare costs associated with extended inpatient care. However, ICA is associated with higher intraoperative costs, as stapling can be more expensive than suturing [19, 20].

Despite ICA’s benefits, it is notable that ICA was predominantly associated with robotic approaches, with a significantly higher proportion of ICA performed robotically in our study and in the literature [21–25]. This correlation may reflect the technical advantages of robotic assistance, which facilitates intracorporeal suturing and stapling through enhanced dexterity and visualization. Although robotic surgery offers these advantages, it also incurs higher costs and longer setup times, factors that need to be balanced against the postoperative benefits of ICA.

Overall, the findings from our study, alongside extensive literature, suggest that ICA should be considered the preferred anastomotic approach for minimally invasive right hemicolectomy with CME, particularly within ERAS frameworks. Given its association with improved postoperative outcomes - including reduced SSI rates, faster recovery, and lower pain levels - ICA presents a compelling case for integration into standard practice. While robotic systems may enhance the feasibility and advantages of ICA, cost and accessibility considerations remain crucial, particularly in healthcare settings with limited resources.

## Conclusion

In conclusion, this study aligns with a growing body of evidence supporting ICA as a potentially superior anastomosis technique for minimally invasive right hemicolectomy with CME. Our findings and those of similar studies underscore ICA’s advantages in postoperative recovery, reduced SSI rates and lower pain levels.

## Data Availability

No datasets were generated or analysed during the current study.

## Declarations

### Statement on the welfare of animals

This article does not contain any studies with animals performed by any of the authors.
